# Isolation, characterization and genes expression analysis of three dehydrin genes during cold acclimation of *Eucalyptus globulus*

**DOI:** 10.1186/1753-6561-5-S7-P81

**Published:** 2011-09-13

**Authors:** Marta Fernández, Sofia Valenzuela

**Affiliations:** 1 Genómica Forestal S.A., Chile; 2 Universidad de Concepción, Facultad de Ciencias Forestales. Centro de Biotecnología. Genómica Forestal S.A., Chile

## 

*Eucalyptus globulus* is an important species for pulp production in Chile; however it has a high sensitivity to frost. During the last few years, many studies have directed their efforts to elucidate the molecular mechanisms that regulate plant response to cold stress through the analysis of gene expression.This work reports the isolation and characterization of coding and non coding sequence of three dehydrin genes of *E. globulus* which allowed comparing the gene expression of these genes during cold acclimation, the type of dehydrin and the presence of regulatory elements in their promoters which provided valuable information about the possible signaling pathways and regulation of these genes. The three dehydrins identified in *E. globulus* showed a high transcript accumulation in stem and leaf tissue of acclimated plants, compared to non-acclimated, and the highest transcript accumulation was observed after the exposition of plants to night frosts (-2°C). Furthermore, the freezing resistant genotype exhibited a higher transcript accumulation after frost exposition compared to the sensitive genotype. These results support the idea that dehydrin proteins have an important rol during cold acclimation and frost tolerance in *E globulus*.

